# Effect of Slit/Robo signaling on regeneration in lung emphysema

**DOI:** 10.1038/s12276-021-00633-8

**Published:** 2021-05-25

**Authors:** Jin-Soo Park, RyeonJin Cho, Eun-Young Kang, Yeon-Mok Oh

**Affiliations:** 1 Asan Institute for Life Sciences, Seoul, Korea; 2 Department of Internal Medicine, University of Ulsan College of Medicine, Seoul, Korea; 3 Department of Pulmonary and Critical Care Medicine, Asan Medical Center, Seoul, Korea

**Keywords:** Molecular biology, Respiratory tract diseases

## Abstract

Emphysema, a pathological component of chronic obstructive pulmonary disease, causes irreversible damage to the lung. Previous studies have shown that Slit plays essential roles in cell proliferation, angiogenesis, and organ development. In this study, we evaluated the effect of Slit2 on the proliferation and migration of mouse lung epithelial cells and its role in regeneration in an emphysema lung mouse model. Here, we have shown that Slit2/Robo signaling contributes to the regeneration of lungs damaged by emphysema. Mouse epithelial lung cells treated with Slit2 exhibited increased proliferation and migration in vitro. Our results also showed that Slit2 administration improved alveolar regeneration in the emphysema mouse model in vivo. Furthermore, Slit2/Robo signaling increased the phosphorylation of ERK and Akt, which was mediated by Ras activity. These Slit2-mediated cellular signaling processes may be involved in the proliferation and migration of mouse lung epithelial cells and are also associated with the potential mechanism of lung regeneration. Our findings suggest that Slit2 administration may be beneficial for alveolar regeneration in lungs damaged by emphysema.

## Emphysema: Can lung cells regenerate?

A protein called Slit2 may help in treating emphysema by promoting regeneration of cells that line the tiny air sacs called alveoli in the lungs. In the alveoli, oxygen is transferred from the lungs to the blood. In emphysema, the alveoli become damaged, reducing patients’ ability to exhale ‘old’ air and limiting capacity to breathe in fresh, oxygen-rich air. No treatments are available. Yeon-Mok Oh at the University of Ulan College of Medicine, Seoul, South Korea and co-workers investigated whether Slit2, known to be involved in nervous system development, cell proliferation, and migration, could aid in regenerating lung cells. Testing in mouse lung cells produced increased proliferation and migration. Further testing in a mouse model of emphysema showed that the alveoli could regenerate. These results hold promise for developing new treatments for emphysema.

## Introduction

Chronic obstructive pulmonary disease (COPD) is characterized by both chronic bronchiolitis and emphysema, with a high global rate of morbidity and mortality^1,2^. To target chronic bronchiolitis, various medications, such as bronchodilators or those with anti-inflammatory effects, have been developed over the past 20 years. However, no treatment has thus far been capable of successfully inducing regeneration in lungs with emphysema, which damages lung alveoli. Slit/Robo signaling, a well-known mediator of nervous system development^3,4^, has also been implicated in cell proliferation, stem cell regulation, angiogenesis, and organ development^5–9^. Recent research has also indicated that Slit/Robo signaling could have important functions in the reproductive system^10^.

Slits are secreted extracellular matrix proteins that bind to the Roundabout (Robo) receptor^11,12^. Three Slit protein isoforms (Slit1–3) have been identified in vertebrates and are characterized by four leucine-rich repeat domains, seven to nine epidermal growth factor repeat domains, a laminin G domain, and a C-terminal cysteine-rich domain^3,13,14^.

Four Robo receptors (Robo1–4) consist of five immunoglobulin (Ig)-like domains with three fibronectin (FN) type III-like domains, a single transmembrane domain, and a long 457 amino acid cytoplasmic tail^13–15^.

Given its role in neurogenesis, cell proliferation, and migration, we hypothesized that Slit/Robo signaling may facilitate regeneration in lungs damaged by emphysema. To test this hypothesis, we evaluated the regenerative effects of the Slit protein in both mouse lung epithelial cells and an elastase-induced emphysema mouse model. Cell proliferation and migration were increased in lung epithelial cells treated with Slit2. Histological analysis and measurement of lung emphysema severity with mean linear intercepts (MLIs) showed that intranasal administration of Slit2 improved lung alveolar regeneration in the emphysema mouse model. These results suggest that Slit2 may have the potential as a new drug candidate for the regeneration of lungs damaged by emphysema.

## Materials and methods

### Epithelial cell proliferation and migration in vitro

Cell proliferation analysis was performed on mouse lung epithelial (MLE-12) cells using Cell Counting Kit-8 (CCK-8, Enzo). MLE-12 cells were seeded on cell culture plates and treated with different concentrations of recombinant murine Slit2 protein (Slit2, R&D Systems). After each time point, proliferation was measured by CCK-8 assay in accordance with the manufacturer’s protocol.

Cell migration was analyzed using a scratch wound healing assay, which involved using a T1000 pipette tip on monolayers of MLE-12 cell cultures. Cells were then incubated in culture media with 50, 100, 200, and 500 ng Slit2 at 37 °C. After 0, 72, and 120 h of incubation, the gap width of the repopulated scratch was measured and recorded and then compared with the initial gap size at 0 h.

### Quantitative real-time polymerase chain reaction (RT-PCR)

Total RNA was isolated from cells or whole-lung tissue using the RNeasy Plus Mini Kit (Qiagen) and synthesized into cDNA using a Maxima First Strand cDNA synthesis kit (Thermo Scientific). Quantitative RT-PCR analyses were performed with a real-time LightCycler 480 and SYBR Green I Master mix.

### Mice

Female C57BL/6 mice, aged 7 weeks, were purchased from Orient Bio (Seongnam, Korea) and maintained under specific pathogen-free conditions in the animal facility of the Asan Institute for Life Sciences. This experiment was approved by the Institutional Animal Care and Use Committee of Asan Medical Center (IACUC. 2017-13-274).

### Elastase-induced lung emphysema model

For the experimental emphysema model, we generated elastase-induced model mice using previously described methods^16^. The mice were intratracheally injected with porcine pancreatic elastase (PPE, Sigma-Aldrich, St. Louis, MO, USA) on day 0. The mice were treated with Slit2 by intranasal administration on days 7–13 and were sacrificed on day 14, after which the lungs were harvested.

### Histology and quantification of emphysema

Lung tissue was inflated with 0.5% low-melting agarose, fixed with 4% formalin, and embedded in paraffin. Lung sections with a thickness of 6 μm were stained with hematoxylin and eosin. The severity of emphysema was measured by MLIs. The MLIs of the lung tissue sections were determined using previously described methods^17^.

### Slit2/Robo binding ELISA

A 96-well microplate was coated with 50 μL of 10 μg of Slit2 or bovine serum albumin (BSA) per well and incubated overnight at 4 °C. The coated plate was then blocked with 100 µL of phosphate-buffered saline (PBS) containing 1% BSA per well for 1 h at room temperature. The plates were washed three times with 200 μL of 0.05% Tween-20 diluted in PBS. MLE-12 cell lysates (100 µL/well) were added to the coated wells, and the plates were incubated for 1 h at room temperature. After being washed, the Robo antibody (1:1000) was added to the 96-well microplates and incubated for 1.5 h at room temperature. After being washed, horseradish peroxidase (HRP)-conjugated secondary antibody was added and incubated for 1 h at room temperature. TMB (3,3′,5,5′-tetramethylbenzidine) substrate solution was added to the 96-well microplates to induce a color reaction. The reaction was then stopped with a stop solution (0.18 M sulfuric acid). The plate was measured at 405 nm on a microplate reader (BioRad).

### siRNA transfection

Robo1 siRNA (Bioneer) was transfected into MLE-12 cells with 100 nmol siRNA in a 6-well plate with Lipofectamine RNAiMax (Invitrogen) according to the manufacturer’s instructions. After 4 h of transfection, the cell culture media was replaced with fresh media, and the cells were used for experiments 48 h after transfection.

### GTPase activity assay

For active GTPase analysis, MLE-12 cells were incubated alone or with Slit2, and the cells were collected at 15 m, 1 h, and 6 h. To harvest the cells, the media was removed, and cells were rinsed with cold PBS. The cells were then resuspended in 0.5 mL of ice-cold 1× lysis/binding/wash buffer plus 1 mM phenylmethylsulfonyl fluoride, and the active guanosine-5′-triphosphate GTP-bound form of proteins was isolated and analyzed according to the manufacturer’s instructions (Cell Signaling Technology). Cell lysates were briefly incubated with glutathione resin and glutathione-S-transferase fusion proteins, and GTP-bound proteins were eluted. The eluted samples were analyzed by Western blotting using antibodies against Ras, Rac1, and Cdc42 (Cell Signaling Technology).

### Western blotting

MLE-12 cells were incubated with or without Slit2 and then collected at 15 m, 1 h, and 6 h by centrifugation at 1200 rpm for 5 m at 4 °C.

Cells were lysed in RIPA buffer and quantified using the BCA assay. Protein samples were separated by sodium dodecyl sulfate-polyacrylamide gel electrophoresis and then transferred to a PVDF membrane. The transferred membranes were incubated at 4 °C overnight with biotinylated primary antibodies against Akt, p-Akt, ERK, p-ERK, and β-actin, incubated with HRP-conjugated secondary antibodies, and detected with a chemiluminescent membrane substrate.

## Results

### Slit2 improves the proliferation and migration of lung epithelial cells in vitro

**Fig. 1:**
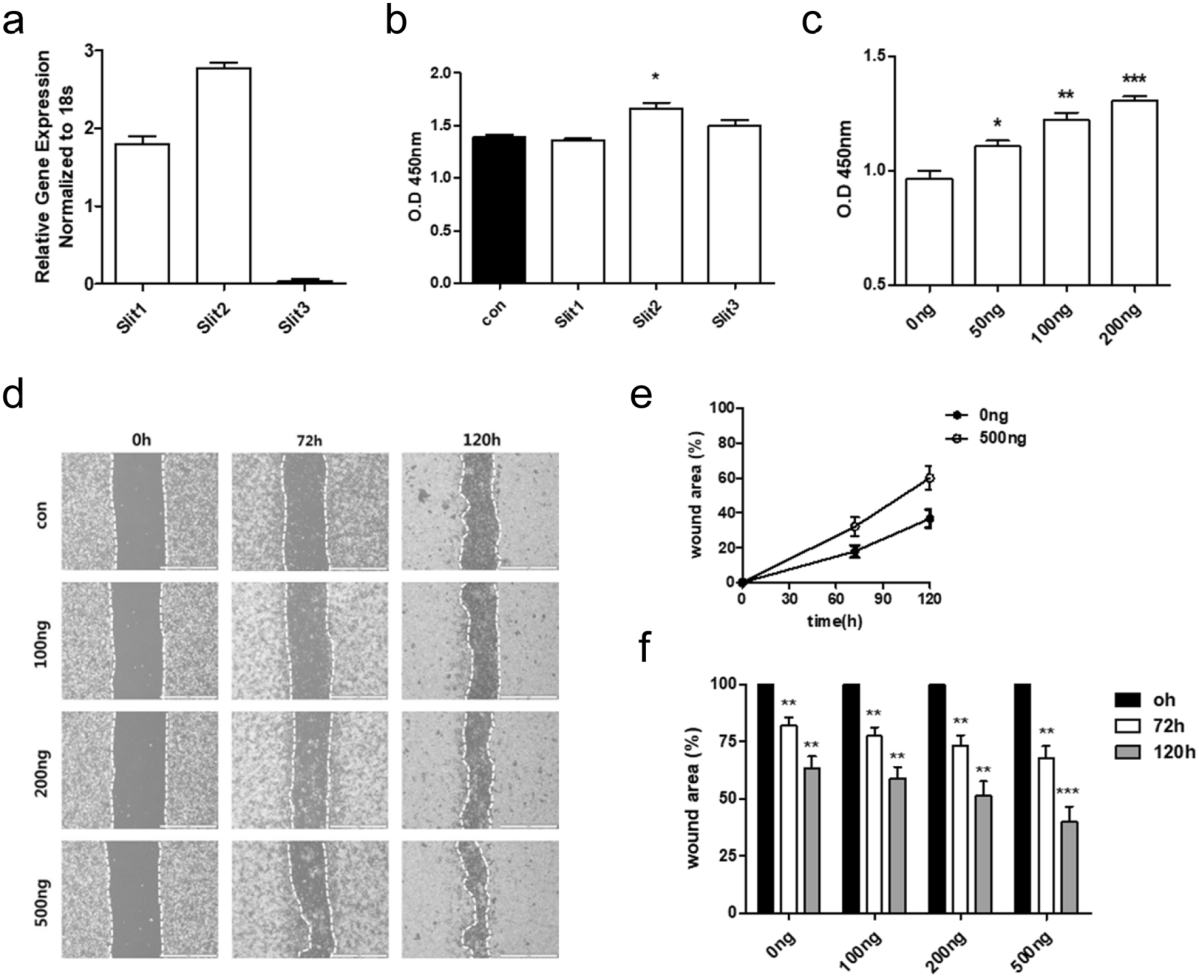
The effects of Slit1–Slit3 on mouse lung epithelial cells (MLE-12 cells) in vitro. **a** Quantitative RT-PCR measurement of Slit levels in mouse lung epithelial (MLE-12) cells. **b** MLE-12 cells were cultured in conditioned media with Slit protein. The proliferation of mouse lung epithelial cells was measured with a CCK-8 assay. **c** MLE-12 cells were treated with different concentrations of Slit2. The proliferation of MLE-12 cells was measured with a CCK-8 assay. **d** A straight scratch was made on MLE-12 cells using a pipette tip. The cells were then cultured with different concentrations of Slit2. **e** The graph shows the % wound healing of MLE-12 cells with or without Slit2 treatment. **f** Treatment with Slit2 promoted the wound healing response after 72 and 120 h. Each experiment was repeated at least three times. Statistically significant differences are indicated. (*p < 0.05, **p < 0.01, ***p < 0.001).

First, we examined the expression pattern of Slit in MLE-12 cells. Slit1 and Slit2 were expressed in MLE-12 cells, whereas Slit3 exhibited little or no expression (Fig. 1a). The protein levels of the Slit family showed similar expression patterns to those in the RT-PCR results (Supplemental Fig. 1). We then examined the effect of Slit 1–3 on MLE-12 cell proliferation in vitro using Slit as a ligand. When MLE-12 cells were treated with each Slit isoform, MLE-12 cells treated with Slit2 exhibited significantly increased proliferation compared to those treated with the other isoforms (Fig. 1b). Next, when Slit2 was added at different concentrations, we found that cell proliferation increased in a concentration-dependent manner (Fig. 1c). The effect of Slit2 on MLE-12 cell migration in vitro was evaluated by using a scratch wound assay. The migration rates were determined by measuring wound closure at defined time points in microscopy images (Fig. 1d). After treatment with Slit2, the recovered wound area confirmed that cell migration more than doubled, and Slit2 improved the movement of MLE-12 cells in a dose-dependent manner (Fig. 1e, f). These data confirmed the expression of Slit in MLE-12 cells, and cell proliferation and migration were increased by Slit2 treatment.

### Expression of the Slit2 receptor and identification of receptor binding

**Fig. 2:**
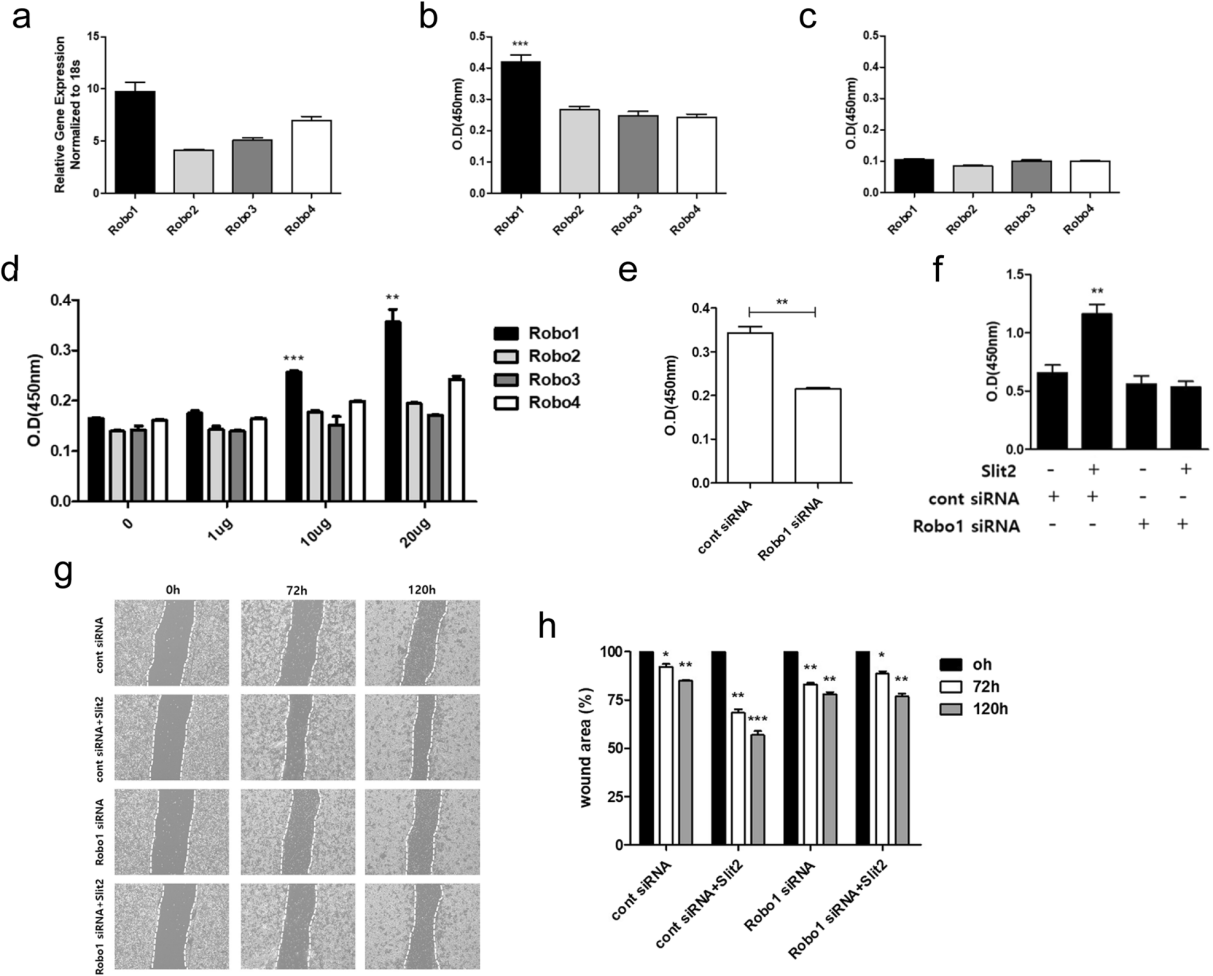
Identification of Slit/Robo signaling. **a** Quantitative RT-PCR measurement of Robo levels in MLE-12 cells. **b**, **c** ELISA binding analysis of Slit2 (**b**) or BSA (**c**) with Robo1–4 in MLE-12 cell lysates. **d** Identification of Robo receptor binding in increasing MLE-12 cell lysate concentrations. **e** Reduced binding of Robo1 to MLE-12 cell lysates (20 µg per lysate) after transfection with siRNA targeting Robo1 or with control siRNA (cont siRNA). **f** MLE-12 cells were transfected with siRNA targeting Robo1 or control siRNA (cont siRNA), and cell proliferation was examined. **g** MLE-12 cells were transfected with siRNA targeting Robo1 or cont siRNA. A straight scratch was made in the MLE-12 cells using a pipette tip. The cells were then cultured with or without Slit2. **h** The recovered wound area in (**g**) was evaluated using ImageJ software. Each experiment was repeated at least three times. Statistically significant differences are indicated. (*p < 0.05, **p < 0.01, ***p < 0.001).

The Slit receptor includes four different types of Robo, and among them, the Robo receptor to which Slit2 preferentially binds was identified. To confirm the expression of Robo receptors, we examined the transcript level of Robo in MLE-12 cells. Overall, the expression of Robo1–4 in MLE-12 cells was confirmed, and the expression of Robo1 was more predominant than that of the others (Fig. 2a). In addition, it was confirmed that the protein level of Robo1 was the highest compared to that of the other proteins (Supplemental Fig. 1). These results suggest that Slit2 will preferentially bind to Robo1. ELISA indicated that the binding of Slit2 and Robo1 was the highest and confirmed that Slit2/Robo1 binding increased with increasing concentrations of MLE-12 cell lysate (Fig. 2b, d). However, BSA and MLE-12 cell lysates did not have a strong affinity for each other (Fig. 2c).

To test the effect of Robo1 knockdown on MLE-12 cells, we applied Robo1 siRNA to MLE-12 cells and silenced the expression of Robo1. With the knockdown of Robo1, the binding of Slit2 to Robo1 in MLE-12 cell lysates was decreased compared to that in the group treated with the control siRNA (Fig. 2e). In MLE-12 cells with Robo1 knockdown, treatment with Slit2 did not affect cell proliferation (Fig. 2f). Furthermore, Robo1 knockdown decreased the migration of MLE-12 cells, as shown by the decrease in the recovered wound area (Fig. 2g, h). These results suggest that Slit2 preferentially binds to Robo1 and that Slit2/Robo1 signaling may have an important effect on the proliferation and migration of MLE-12 cells.

### Regenerative effects of Slit2 on an elastase-induced emphysema mouse model

**Fig. 3:**
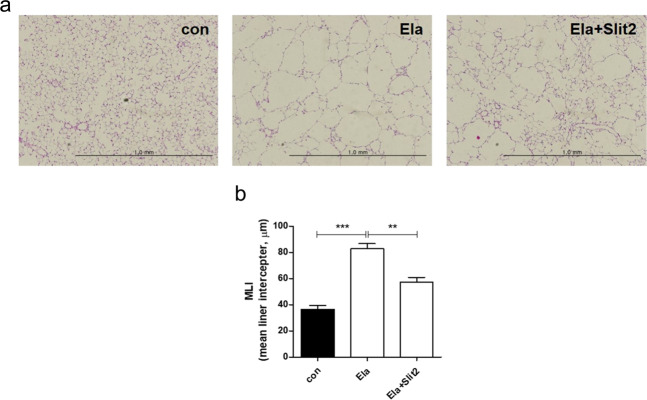
The effects of Slit2 on lung regeneration in mice with elastase-induced emphysema. **a** C57BL/6 mice were intratracheally injected with elastase (Ela) on day 0 and then intranasally administered Slit2 (500 ng) on days 7–13. Hematoxylin and eosin (H&E)-stained lung tissue sections on day 14. **b** Morphometric analysis of the mean linear intercept (MLI). The values represent the mean±standard error of the mean (*n* = 6). Statistically significant differences are indicated. (**p < 0.01, ***p < 0.001).

We confirmed that Slit2 is involved in MLE-12 cell proliferation and migration in vitro, and we tested the hypothesis that Slit2 plays a role in lung regeneration in the elastase-induced emphysema mouse model. After inducing emphysema with elastase, mice were treated by daily intranasal administration of Slit2 from days 7–13. To determine whether Slit2 restores elastase-induced lung destruction, lung tissue sections were stained with hematoxylin and eosin to analyze the histological changes in MLI counts. Alveolar damage and MLI increases were indicated in the group treated with elastase alone, and lung regeneration and MLI reductions were indicated in the group administered Slit2 after elastase injection (Fig. 3a, b). These data indicated that Slit2 administration may have a regenerative effect on the elastase-induced emphysema mouse model.

### Slit2 activates signaling pathways involved in cellular processes

**Fig. 4:**
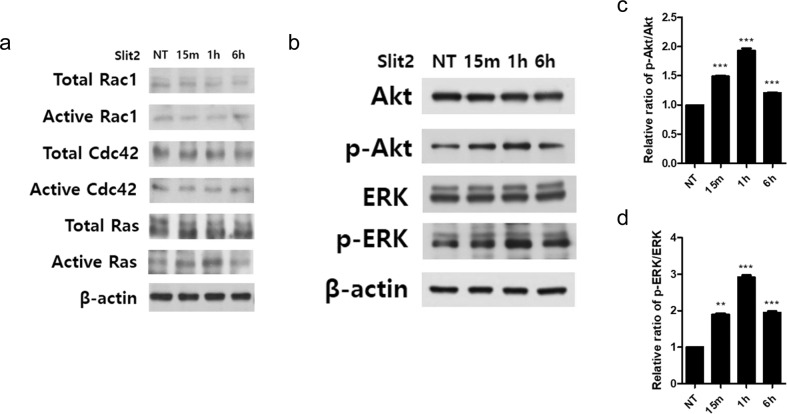
The signal transduction of Slit2 in MLE-12 cells. **a** Western blot analysis demonstrating the levels of active and total GTPases (Rac1, Cdc42, and Ras) and actin in MLE-12 cells with or without Slit2 treatment (NT). **b** Western blot analysis demonstrating the levels of Akt and ERK phosphorylation in MLE-12 cells with or without Slit2 treatment. **c**, **d** Quantification of the phosphorylation level was determined in the same manner as in (**b**) using ImageJ software. The results represent at least three independent experiments. Statistically significant differences are indicated. (**p < 0.01, ***p < 0.001).

It is widely known that Slit/Robo signaling involves GTPases, which are small GTP-binding proteins that include the Ras superfamily: Ras, Rho, Rab, Arf, and Ran^18–21^. In addition, these proteins may be involved in regulatory mechanisms such as cell migration, angiogenesis, and neuronal morphogenesis^22–24^. We then examined whether Slit2 treatment increased the activity of GTPases in MLE-12 cells. Slit2 increased the levels of GTP-bound Ras but not the GTPases in the Rho family (Rac1 and Cdc42) (Fig. 4a). When bound to GTP, Ras is activated to stimulate multiple downstream targets, such as the ERK/MAP kinase pathway and PI3-kinase/Akt^25–27^. The ERK/MAP kinase and PI3-kinase/Akt pathways are major intracellular signaling pathways that are known to regulate various cellular processes, including cell proliferation, migration, and survival^27–30^. We, therefore, aimed to investigate whether ERK and Akt signal transduction was involved in Slit2-induced cell proliferation and migration. We examined the effect of Slit2 on the phosphorylation of ERK and Akt in MLE-12 cell lysates after Slit2 treatment. As shown in Fig. 4, Slit2 activated ERK and Akt phosphorylation. Phosphorylation increased within 15 min of Slit2 administration and peaked at 1 h (Fig. 4b). Densitometric analysis was performed to determine the ratio of phosphorylated to total ERK and Akt (Fig. 4c, d). When Robo1 was knocked down, there was no change in Ras activity or the phosphorylation of ERK and Akt by slit2 compared to the effects of control siRNA (Supplemental Fig. 2).

The results showed that Slit2-induced GTP-bound Ras increased phosphorylation of the downstream targets ERK and Akt and thus affected the proliferation and migration of MLE-12 cells.

## Discussion

Recent studies have shown that Slit/Robo signaling has been implicated in cell proliferation, stem cell regulation, angiogenesis, and organ development^4,6,8,9^. Slit proteins are secreted glycoproteins with three isoforms; these proteins are involved in regulating the migration of axons and neurons during development^13,14^. Slit proteins bind to four Robo receptors (Robo1–Robo4), which are associated with downstream signaling that regulates various cellular processes^11,31^.

In this study, we examined the regenerative effect of Slit2 on cell proliferation and migration and an elastase-induced emphysema mouse model. Our findings showed that Slit2 improved MLE-12 cell proliferation and migration, which was associated with Slit2 signaling through Robo1. In the elastase-induced emphysema mouse model, intranasal administration of Slit2 showed therapeutic effects on the regeneration of alveolar destruction. Slit2 treatment increased GTP-binding to Ras, which increased phosphorylation of the downstream targets ERK and Akt and may affect the proliferation and migration of MLE-12 cells. Research related to Slit has mainly focused on the nervous system, such as the regulation of axon and neuronal migration during development; however, Slit was recently confirmed to affect bone regeneration^32^ and was found to play an important role. Slit/Robo signaling has also been shown to regulate the vascular system, including angiogenic processes such as endothelial cell migration and tube formation^33^.

With these studies as a foundation for our research, we present for the first time results that indicate that Slit2 may be effective for lung regeneration.

There is currently no standard treatment regimen for COPD; it is, therefore, vital to develop a therapeutic agent, and Slit2 shows potential as a treatment candidate based on its regenerative effects. Here, we first focused on the proliferation of alveolar epithelial cells in vitro and found that Slit2 could increase the proliferation of these cells. Slit and Robo each have several isoforms. Our data showed that compared with the other Slit proteins, Slit2 increased the proliferation and migration of MLE-12 cells, and of the Robo protein family, Robo1 preferentially bound to Slit2.

In addition, when Robo1 was knocked down using Robo1 siRNA, the proliferation of MLE-12 cells did not increase in response to Slit2 treatment, suggesting that Slit2/Robo1 signaling may play an important role in lung regeneration.

Next, based on the in vitro results, we investigated whether Slit2 affected lung regeneration.

Recently, there has been an effort to develop a new drug for bone regeneration that targets Slit, which is an important mechanism for bone regeneration^32^. On the other hand, considering that Slit is also present in the lungs, based on the in vitro results, we aimed to confirm whether Slit2 could act on emphysema-associated lung regeneration. When the mouse emphysema model was induced with elastase and intranasally administered Slit2, the therapeutic effects on the repair of alveolar damage were shown. Furthermore, among GTPases, Ras-mediated activation of ERK and Akt was increased by increasing Ras activity through Slit2/Robo signaling.

In summary, these factors may play critical roles in epithelial cell proliferation, facilitating the repair of the damaged epithelium associated with lung regeneration.

Our findings suggest for the first time that Slit2 may have potential as a therapeutic drug candidate for the treatment of emphysema-associated lung regeneration.

## Supplementary information

Supplemental Material File #1
