# Supplementary material for: Effect of Slit/Robo signaling on regeneration in lung emphysema
Source: Exp Mol Med. 2021 May 25;53(5):986–92. doi: 10.1038/s12276-021-00633-8 (PMC8178402; doi:10.1038/s12276-021-00633-8)
Supplement: Supplementary file 1 — Supplemental Material File #1 [file 12276_2021_633_MOESM1_ESM.pdf]

supplementary information

Supplemental figure 1. Expression level of Slit and Robo

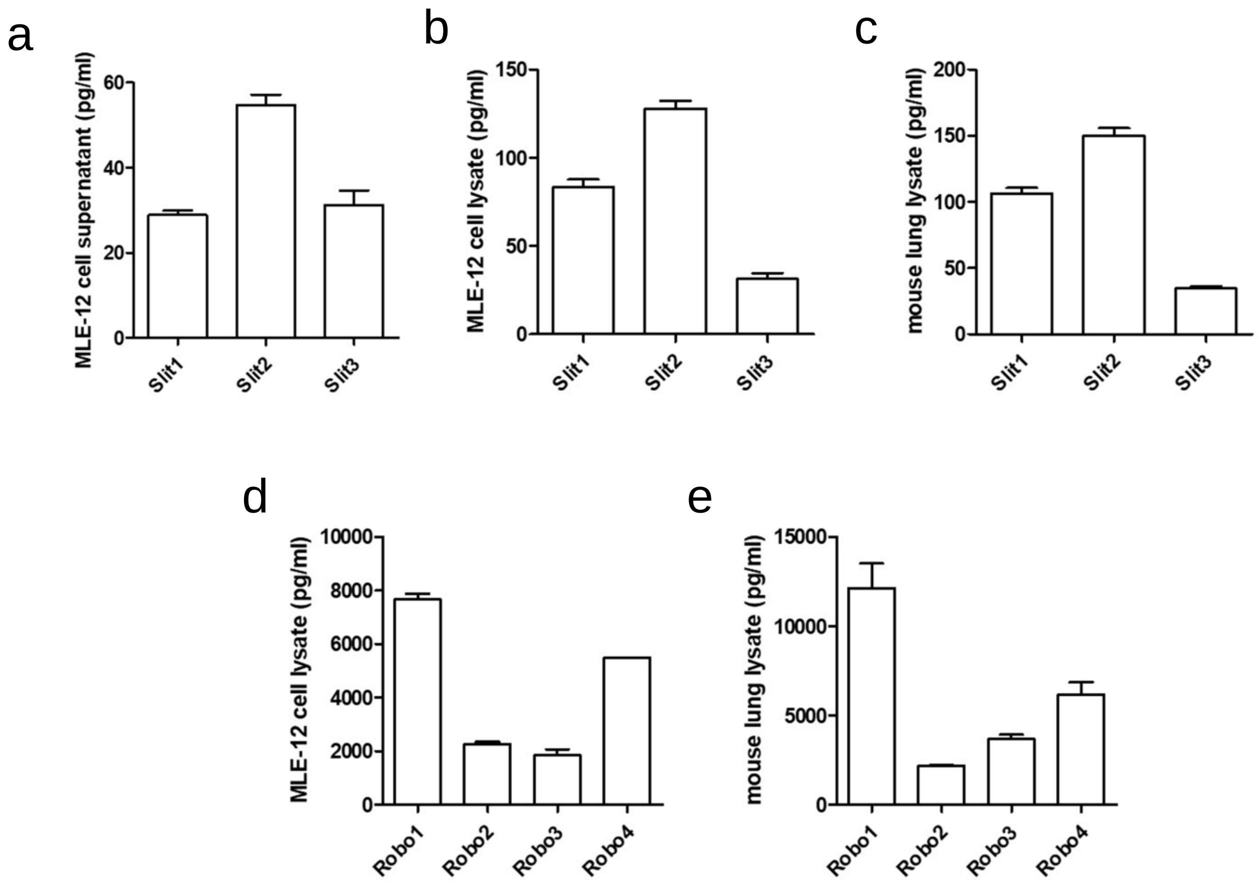

Supplemental figure 2. The signal transduction of Slit2 in MLE-12 cells with Robo1 siRNA

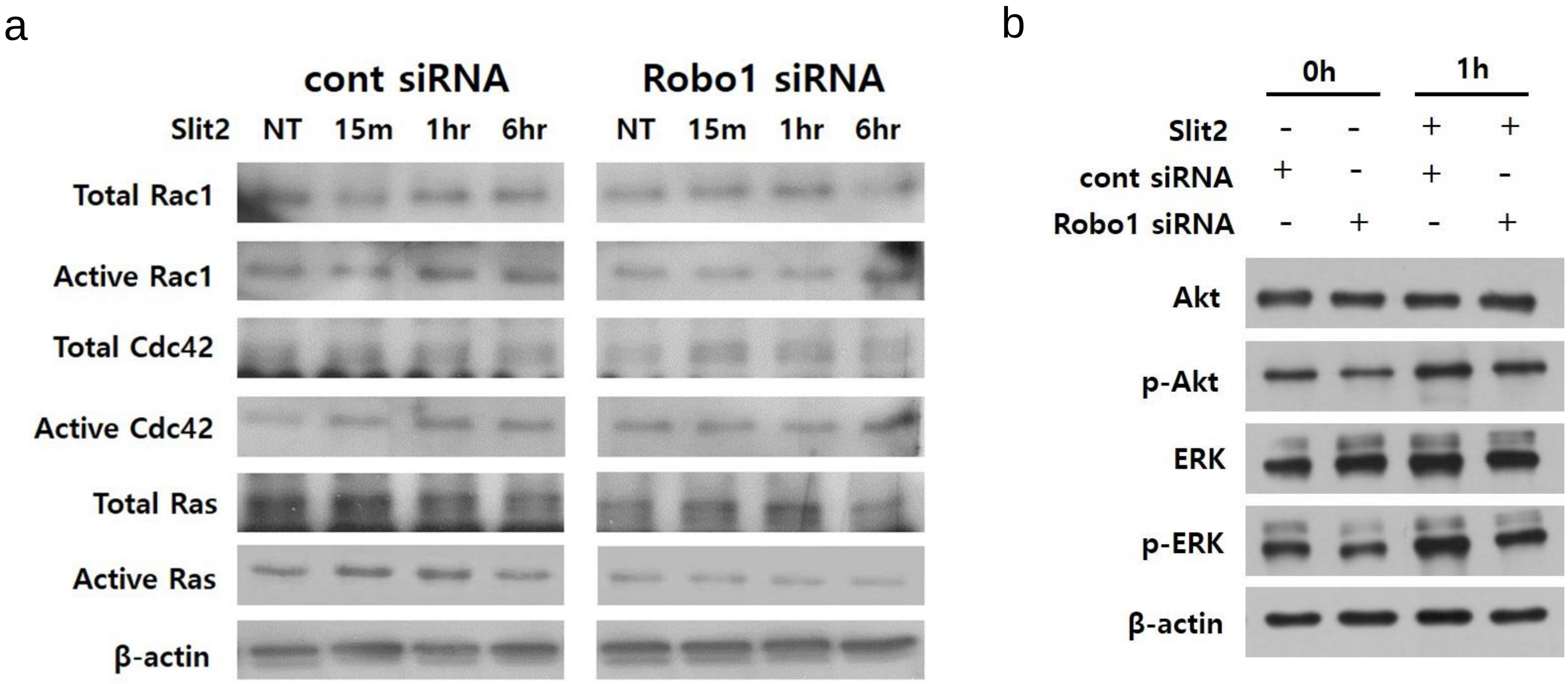

Supplemental Figure 1. Expression level of Slits and Robos

(a, b, c) The protein expression of Slits in MLE-12 cell supernatant, cell lysate and mouse lung tissue. (d, e) The protein expression of Robos in MLE-12 cell lysates and mouse lung tissue.

Supplemental Figure 2. The signal transduction of Slit2 in MLE-12 cells with Robo1 siRNA.

(a) Western blot analysis demonstrating the levels of active and total GTPases (Rac1, Cdc42, Ras) and actin in MLE-12 cells transfected with Robo1 siRNA or control siRNA (cont siRNA). (b) Western blot analysis demonstrating the levels of Akt and ERK phosphorylation in MLE-12 cells transfected with Robo1 siRNA or cont siRNA. The results represent at least three independent experiments.
